# Multimodal imaging and detection approach to ^18^F-FDG-directed surgery for patients with known or suspected malignancies: a comprehensive description of the specific methodology utilized in a single-institution cumulative retrospective experience

**DOI:** 10.1186/1477-7819-9-152

**Published:** 2011-11-23

**Authors:** Stephen P Povoski, Nathan C Hall, Douglas A Murrey, Andrew Z Chow, Jay R Gaglani, Eamonn E Bahnson, Cathy M Mojzisik, Maureen P Kuhrt, Charles L Hitchcock, Michael V Knopp, Edward W Martin

**Affiliations:** 1 Division of Surgical Oncology, Department of Surgery, Arthur G. James Cancer Hospital and Richard J. Solove Research Institute and Comprehensive Cancer Center, The Ohio State University Medical Center, Columbus, Ohio, 43210, USA; 2 Department of Radiology, The Ohio State University Medical Center, Columbus, Ohio, 43210, USA; 3 Veeda Oncology, Columbus, Ohio, 43215, USA; 4 Department of Pathology, The Ohio State University Medical Center, Columbus, Ohio, 43210, USA; 5 Wright Center of Innovation in Biomedical Imaging, The Ohio State University Medical Center, Columbus, Ohio, 43210, USA

## Abstract

**Background:**

^18^F-FDG PET/CT is widely utilized in the management of cancer patients. The aim of this paper was to comprehensively describe the specific methodology utilized in our single-institution cumulative retrospective experience with a multimodal imaging and detection approach to ^18^F-FDG-directed surgery for known/suspected malignancies.

**Methods:**

From June 2005-June 2010, 145 patients were injected with ^18^F-FDG in anticipation of surgical exploration, biopsy, and possible resection of known/suspected malignancy. Each patient underwent one or more of the following: (1) same-day preoperative patient diagnostic PET/CT imaging, (2) intraoperative gamma probe assessment, (3) clinical PET/CT specimen scanning of whole surgically resected specimens (WSRS), research designated tissues (RDT), and/or sectioned research designated tissues (SRDT), (4) micro PET/CT specimen scanning of WSRS, RDT, and/or SRDT, (5) total radioactivity counting of each SRDT piece by an automatic gamma well counter, and (6) same-day postoperative patient diagnostic PET/CT imaging.

**Results:**

Same-day ^18^F-FDG injection dose was 15.1 (± 3.5, 4.6-26.1) mCi. Fifty-five same-day preoperative patient diagnostic PET/CT scans were performed. One hundred forty-two patients were taken to surgery. Three of the same-day preoperative patient diagnostic PET/CT scans led to the cancellation of the anticipated surgical procedure. One hundred forty-one cases utilized intraoperative gamma probe assessment. Sixty-two same-day postoperative patient diagnostic PET/CT scans were performed. WSRS, RDT, and SRDT were scanned by clinical PET/CT imaging and micro PET/CT imaging in 109 and 32 cases, 33 and 22 cases, and 49 and 26 cases, respectively. Time from ^18^F-FDG injection to same-day preoperative patient diagnostic PET/CT scan, intraoperative gamma probe assessment, and same-day postoperative patient diagnostic PET/CT scan were 73 (± 9, 53-114), 286 (± 93, 176-532), and 516 (± 134, 178-853) minutes, respectively. Time from ^18^F-FDG injection to scanning of WSRS, RDT, and SRDT by clinical PET/CT imaging and micro PET/CT imaging were 389 (± 148, 86-741) and 458 (± 97, 272-656) minutes, 619 (± 119, 253-846) and 661 (± 117, 433-835) minutes, and 674 (± 186, 299-1068) and 752 (± 127, 499-976) minutes, respectively.

**Conclusions:**

Our multimodal imaging and detection approach to ^18^F-FDG-directed surgery for known/suspected malignancies is technically and logistically feasible and may allow for real-time intraoperative staging, surgical planning and execution, and determination of completeness of surgical resection.

## Background

^18^F-fluorodeoxyglucose (^18^F-FDG) positron emission tomography/computed tomography (PET/CT) is widely used in the clinical management of cancer patients and has become the cornerstone of diagnostic imaging, staging, follow-up surveillance, and monitoring of ongoing therapy for a wide variety of malignancies [1-7]. In this regard, there has been increased interest and growth in clinical research directed towards the use of ^18^F-FDG for the intraoperative detection of known and occult malignant disease during cancer surgery [8-48]. The application of ^18^F-FDG for intraoperative detection during cancer surgery was first described in 1999 for colorectal cancer [8]. Since that time, a significant portion of this ongoing work related to the use of ^18^F-FDG for intraoperative detection during cancer surgery has been conducted at The Ohio State University and has been directed toward multiple solid malignancies [8-10,22-24,28-33,36,37,40]. Collectively, such efforts have been directed toward colorectal cancer, melanoma, lymphoma, breast cancer, gynecologic malignancies, head and neck malignancies, and lung cancer [8-48]. The motivation behind using ^18^F-FDG for intraoperative detection during cancer surgery has been multifactorial, including exploring its applicability for real-time intraoperative staging, surgical planning and execution, and determination of completeness of surgical resection for ^18^F-FDG-avid lesions.

In 2007, we first reported upon our ongoing efforts to formulate a truly multimodal imaging and detection approach to ^18^F-FDG-directed surgery [23,32,36,40]. In the general schema for this multimodal approach, patients undergoing same-day intravenous administration of ^18^F-FDG were subjected to one or more of a variety of ^18^F-FDG-related diagnostic procedures, including (1) same-day preoperative patient diagnostic PET/CT imaging, (2) intraoperative gamma probe assessment, (3) clinical PET/CT specimen scanning of whole surgically resected specimens (WSRS), research designated tissues (RDT), and/or sectioned research designated tissues (SRDT) (Table 1), (4) micro PET/CT specimen scanning of WSRS, RDT, and/or SRDT, (5) total radioactivity counting of each SRDT piece by an automatic gamma well counter, and (6) same-day postoperative patient diagnostic PET/CT imaging. Herein, we have comprehensively described the specific methodology utilized in our single-institution cumulative retrospective experience with a multimodal imaging and detection approach to ^18^F-FDG-directed surgery for patients with known or suspected malignancies, in order to assess its technical and logistic feasibility for real-time intraoperative staging, surgical planning and execution, and determination of completeness of surgical resection for ^18^F-FDG-avid lesions detected in patients who are deemed as appropriate surgical candidates.

**Table 1:** Tissues collected at the time of biopsy and resection of a known or suspected malignancy at the time of ^18^F-FDG-directed surgery.

Tissue designation	Abbreviation	Description of tissue designation
Whole Surgically Resected Specimen	WSRS	Intact tissue removed as a biopsy specimen or as a resected specimen

Research Designated Tissue	RDT	An approximately 5 mm thick representative portion of tissue from the WSRS which contained both gross tumor and normal appearing tissue

Sectioned Research Designated Tissue	SRDT	Sectioned pieces of tissue that resulted when the RDT was cut into multiple portions of tissue that were of a size that would allow each such sectioned piece of tissue to fit into an individual pathology cassette (i.e., approximately 1.0 cm × 1.0 cm × 0.5 cm)

## Methods

### Patient population

This retrospective review, representing our cumulative experience with a multimodal imaging and detection approach to ^18^F-FDG-directed surgery for known or suspected malignancies from June 2005 to June 2010 at the Arthur G. James Cancer Hospital and Richard J. Solove Research Institute of The Ohio State University Medical Center (OSUMC), was approved by the OSUMC Cancer Institutional Review Board.

All patients evaluated from this retrospective cumulative experience were planned for surgical exploration, biopsy, and possible resection within the operating room of a known or suspected malignancy, based upon the standard of care management for their particular disease entity. All patients evaluated had to have a known or suspected malignancy, as well as had to be ≥ 18 years of age, non-pregnant, and healthy enough to be considered for surgical exploration, biopsy, and possible resection within the operating room. Written informed consent was obtained from all patients on the day of surgery after all aspects of the proposed multimodal approach were fully discussed with them and prior to initiation of any ^18^F-FDG-directed procedures.

### Schema of multimodal imaging and detection approach to ^18^F-FDG-directed surgery

**Figure 1:**
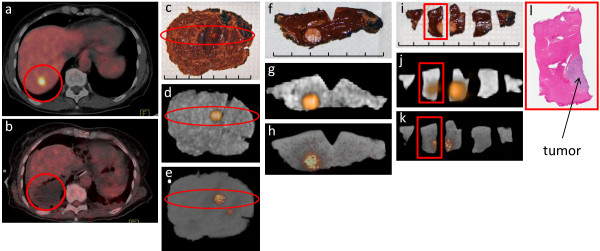
**Segment 7 hepatic metastasis from colorectal cancer**: (a) Preoperative patient diagnostic PET/CT scan demonstrating an ^18^F-FDG-avid lesion in the liver (red circle). (b) Postoperative patient diagnostic PET/CT demonstrating complete removal of the ^18^F-FDG-avid lesion (red circle). (c) Digital photo of the WSRS (i.e., segment 7 liver resection specimen), visualizing the hepatic metastasis (red oval). (d) Clinical PET/CT specimen image and (e) micro PET/CT specimen image of the WSRS, demonstrating the ^18^F-FDG-avid lesion (red oval). (f) Digital photo depicting the first phase of the pathologic processing that produced the RDT, which consists of a single 0.5 cm slice through the hepatic metastasis. (g) Clinical PET/CT specimen image and (h) micro PET/CT specimen image of the RDT, demonstrating the ^18^F-FDG-avid lesion that corresponds to the hepatic metastasis. (i) Digital photo after sectioning of the RDT into five pieces of tissue, designated as SRDT, with two pieces containing visible tumor. (j) Clinical PET/CT specimen image and (k) micro PET/CT image of the SRDT, demonstrating ^18^F-FDG avidity in the two pieces that corresponds to the hepatic metastasis. (l) H&E stained, whole-mount slide (0.4× magnification) of the specific SRDT piece that is highlighted in the red rectangle in (i), (j), and (k), demonstrating histologic confirmation of a colorectal cancer hepatic metastasis and the corresponding location of tumor within this specific SRDT piece. Each division of the hatched line in (c), (f), and (i) represents 1 cm.

**Figure 2:**
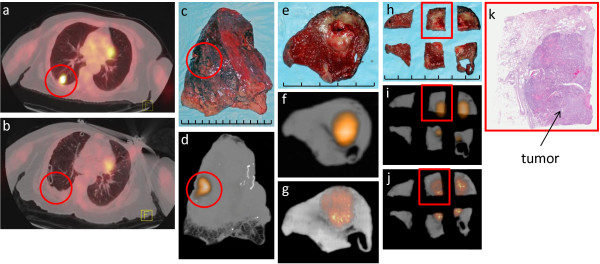
**Right lower lobe lung cancer**: (a) Preoperative patient diagnostic PET/CT scan demonstrating an ^18^F-FDG-avid lesion in the right lower lobe (red circle). (b) Postoperative patient diagnostic PET/CT demonstrating complete removal of the ^18^F-FDG-avid lesion (red circle). (c) Digital photo of the WSRS (i.e. right lower lobectomy specimen), visualizing the lung cancer (red circle). (d) Clinical PET/CT specimen image of the WSRS, demonstrating the ^18^F-FDG-avid lesion (red circle). (e) Digital photo depicting the first phase of the pathologic processing that produced the RDT, which consists of a single 0.5 cm slice through the lung cancer. (f) Clinical PET/CT specimen image and (g) micro PET/CT specimen image of the RDT, demonstrating the ^18^F-FDG-avid lesion that corresponds to the lung cancer. (h) Digital photo after sectioning of the RDT into six pieces of tissue, designated as SRDT, with four pieces containing visible tumor. (i) Clinical PET/CT specimen image and (j) micro PET/CT image of the SRDT, demonstrating ^18^F-FDG avidity in the four pieces that corresponds to the lung cancer. (k) H&E stained, whole-mount slide (0.4× magnification) of the specific SRDT piece that is highlighted in the red rectangle in (h), (i), and (j), demonstrating histologic confirmation of the lung cancer and the corresponding location of tumor within this specific SRDT piece. Each division of the hatched line in (c), (e), and (h) represents 1 cm.

**Figure 3:**
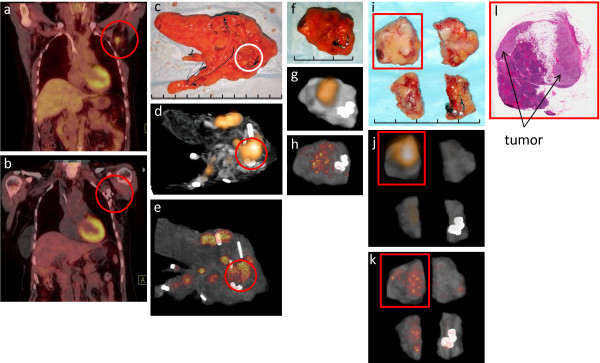
**Left axillary recurrence from breast cancer**: (a) Preoperative patient diagnostic PET/CT scan demonstrating an ^18^F-FDG-avid lesion in the left axilla (red circle). (b) Postoperative patient diagnostic PET/CT demonstrating complete removal of the ^18^F-FDG-avid lesion (red circle). (c) Digital photo of the WSRS (i.e., left axillary lymph node dissection specimen), with the white circle corresponding to a suspicious palpable lymph node that is to be further processed. (d) Clinical PET/CT specimen image and (e) micro PET/CT specimen image of the WSRS, with the red circle demonstrating the ^18^F-FDG-avid lesion that is to be further processed. (f) Digital photo depicting the first phase of the pathologic processing that produced the RDT, which consists of a single 0.5 cm slice through a piece of tissue containing the suspicious palpable lymph node. (g) Clinical PET/CT specimen image and (h) micro PET/CT specimen image of the RDT, demonstrating the ^18^F-FDG-avid lesion that corresponds to the piece of tissue containing the suspicious palpable lymph node. (i) Digital photo after sectioning of the RDT into four pieces of tissue, designated as SRDT, with visible tumor seen within the piece of tissue shown in the red square. (j) Clinical PET/CT specimen image and (k) micro PET/CT image of the SRDT, demonstrating ^18^F-FDG avidity within the piece of tissue shown in the red square that corresponds to the visible tumor within the previously processed portion of the suspicious palpable lymph node. (l) H&E stained, whole-mount slide (0.4× magnification) of the specific SRDT piece that is highlighted in the red square in (i), (j), and (k), demonstrating histologic confirmation breast cancer within the corresponding previously processed portion of the suspicious palpable lymph node. Each division of the hatched line in (c), (f), and (i) represents 1 cm.

**Figure 4:**
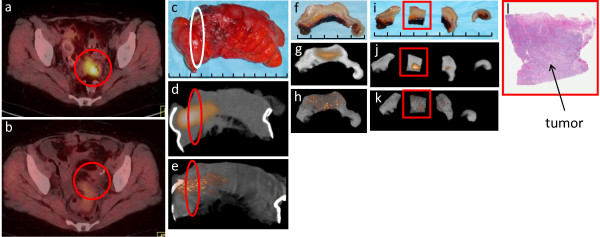
**Rectosigmoid colon recurrence from cervical cancer**: (a) Preoperative patient diagnostic PET/CT scan demonstrating an ^18^F-FDG-avid lesion in the rectosigmoid colon (red circle). (b) Postoperative patient diagnostic PET/CT demonstrating complete removal of the ^18^F-FDG-avid lesion (red circle). (c) Digital photo of the WSRS (i.e., segmental rectosigmoid colon resection specimen), demonstrating the area of the rectosigmoid colon recurrence (white oval). (d) Clinical PET/CT specimen image and (e) micro PET/CT specimen image of the WSRS, demonstrating the ^18^F-FDG-avid lesion (red oval). (f) Digital photo depicting the first phase of the pathologic processing that produced the RDT, which consists of a single 0.5 cm slice through the rectosigmoid colon recurrence. (g) Clinical PET/CT specimen image and (h) micro PET/CT specimen image of the RDT, demonstrating the ^18^F-FDG-avid lesion that corresponds to the rectosigmoid colon recurrence. (i) Digital photo after sectioning of the RDT into four pieces of tissue, designated as SRDT, with two pieces containing visible tumor. (j) Clinical PET/CT specimen image and (k) micro PET/CT image of the SRDT, demonstrating ^18^F-FDG avidity in the two pieces that corresponds to the rectosigmoid colon recurrence. (l) H&E stained, whole-mount slide (0.4× magnification) of the specific SRDT piece that is highlighted in the red square in (i), (j), and (k), demonstrating histologic confirmation of the rectosigmoid colon recurrence of cervical cancer and the corresponding location of tumor within this specific SRDT piece. Each division of the hatched line in (c), (f), and (i) represents 1 cm.

For this multimodal schema, patients underwent one or more of a variety of ^18^F-FDG-related diagnostic procedures, including (1) same-day preoperative patient diagnostic PET/CT imaging, (2) intraoperative gamma probe assessment, (3) clinical PET/CT scanning of whole surgically resected specimens (WSRS), research designated tissues (RDT), and/or sectioned research designated tissues (SRDT) (Table 1), (4) micro PET/CT scanning of WSRS, RDT, and/or SRDT, (5) total radioactivity counting of each SRDT piece by an automatic gamma well counter, and (6) same-day postoperative patient diagnostic PET/CT imaging. Four examples of this multimodal imaging and detection approach to ^18^F-FDG-directed surgery are shown in Figures 1, 2, 3, and 4. Various components of this schema have been previously described [23,32].

In general, patients underwent whole body diagnostic PET/CT scanning either prior to the date of their ^18^F-FDG-directed surgery or on the day of their ^18^F-FDG-directed surgery. The ^18^F-FDG dosing used for all whole body diagnostic PET/CT scans was based upon standard-of-care practice guidelines recommended in the United States [49,50]. All such whole body diagnostic PET/CT scans were reviewed and interpreted by a board certified nuclear medicine physician and a surgical oncologist prior to their ^18^F-FDG-directed surgery. During the time frame of this retrospective, cumulative experience at OSUMC, three different diagnostic PET/CT imaging units were utilized, and included: (1) Siemens Biograph 16 (Siemens, Knoxville, Tennessee, (2) Phillips Gemini TF (Philips, Amsterdam, Netherlands), and (3) Siemens Biograph mCT (Siemens, Knoxville, Tennessee).

On the date of the anticipated ^18^F-FDG-directed surgery, all patients were intravenously injected with ^18^F-FDG, based upon standard-of-care practice guidelines recommended for ^18^F-FDG dosing for whole body diagnostic PET/CT scanning in the United States [49,50]. After receiving their same-day ^18^F-FDG injection, patients either: (1) underwent a same-day preoperative patient diagnostic PET/CT scan and were then taken to the preoperative holding area before proceeding to the operating room for their ^18^F-FDG-directed surgery or (2) were directly taken to the preoperative holding area before proceeding to the operating room for their anticipated ^18^F-FDG-directed surgery.

Intraoperatively, potential sites of tumor were attempted to be localized by classical surgical techniques of visual inspection and palpation. Thereafter, potential sites of tumor were attempted to be localized by intraoperative gamma probe assessment. During the time frame of this retrospective, cumulative experience, three different handheld gamma detection probe systems were utilized, and included: (1) Neoprobe 1000^® ^(Neoprobe Corporation, Dublin, Ohio), (2) Neoprobe 2000^® ^(Neoprobe Corporation, Dublin, Ohio), and (3) RMD Navigator™ GPS (RMD Instruments Corp, Watertown, Massachusetts).

The surgeon then proceeded with biopsy and possible resection of the known or suspected malignancy, based upon the standard of care management for the particular disease entity. Whole surgically resected specimens (WSRS), once removed by the surgeon, were computer documented and accessioned by the operating room nursing staff, and were then taken directly to the Pathology Department for initiation of specimen processing. As per hospital policy, assignment of a pathology accession number and gross examination of each specimen was performed and documented by a surgical pathology technologist.

When technically feasible, WSRS were then taken to the PET/CT suite of the Radiology Department and mounted on a rectangular one-half inch thick slab of paraffin. Digital photographs of the WSRS were taken to document visible appearance of the WSRS with the appearance of the WSRS on clinical PET/CT specimen imaging and/or micro PET/CT specimen imaging. PET/CT imaging was then performed on the WSRS using the same diagnostic PET/CT imaging unit that was used for the same-day preoperative patient diagnostic PET/CT scan, if performed. Images were processed and reviewed for quality and for determination of the presence or absence of ^18^F-FDG-avid foci. This was then correlated to the ^18^F-FDG-avid foci originally seen on the whole body diagnostic PET/CT scan done prior to the date of their ^18^F-FDG-directed surgery or on the day of their ^18^F-FDG-directed surgery. In selected cases, micro PET/CT specimen imaging was then performed on WSRS utilizing the Inveon micro PET/CT scanner and Inveon Acquisition Workplace (Siemens Medical Solutions, Knoxville, Tennessee), if the WSRS were small enough to fit into the 12 cm bore of the micro PET/CT scanner unit.

The WSRS was then returned to the Pathology Department for further specimen processing. In selected cases, from the WSRS, an approximately 5 mm thick representative research designated tissue specimen was harvested which contained both gross tumor and normal appearing tissue and was defined as research designated tissues (RDT). This 5 mm thickness was chosen to accommodate the depth of a standard pathology cassette. The RDT was returned to the PET/CT suite of the Radiology Department, digitally photographed to aid in localization and orientation, and again underwent clinical PET/CT specimen imaging and/or micro PET/CT specimen imaging described above for the WSRS.

In selected cases, the RDT was then cut into multiple pieces of a size that would allow each such piece to fit into individual pathology cassettes (i.e., approximately 1.0 cm × 1.0 cm × 0.5 cm). The resultant pieces of tissue were designated as sectioned research designated tissues (SRDT). These cuts were made such that at least one SRDT piece contained a visible transition from grossly visible tumor to grossly normal appearing tissue. The SRDT pieces were digitally photographed to document the location and orientation of each SRDT piece as it related to the previous RDT. Each side of an individual SRDT piece was inked in a different color for later identifying the original orientation of each SRDT piece upon microscopy at histologic analysis after later tissue processing, sectioning and H&E staining. As with the WSRS and RDT, the SRDT pieces underwent clinical PET/CT specimen imaging and/or micro PET/CT specimen imaging. Each SRDT piece was then individually weighed and placed into an automatic gamma well counter (1282 Compugamma CS, LKB Wallac Corporation, Turku, Finland) to determine the total radioactivity within each SRDT piece. Each SRDT piece was then placed into its own individual pathology cassette, labeled such that its orientation could be maintained, and returned to the Pathology Department for standard processing.

At the completion of the ^18^F-FDG-directed surgery, patients were taken to the postanesthesia care unit for standard postoperative recovery. Thereafter, if determined to be medically safe, patients were transported to the PET/CT suite of the Radiology Department for same-day postoperative patient diagnostic PET/CT imaging utilizing the same-day ^18^F-FDG injection given earlier in the day and with no additional ^18^F-FDG administered. The same-day postoperative patient diagnostic PET/CT scan was performed in a similar fashion as the same-day preoperative patient diagnostic PET/CT scan, using the same imaging unit, but reducing the field of imaging to the region of the body where the ^18^F-FDG-avid foci were originally located on the preoperative patient diagnostic PET/CT scan.

All standard precautions were followed regarding radiation safety and monitoring, and were in compliance with the regulatory standards and policies set forth by OSUMC Radiation Safety Program of The Office of Environmental Health and Safety for radionuclide administration, and have been previously described [33].

### Statistical analysis

Results were expressed as mean (± SD, range). The software program IBM SPSS^® ^19 for Windows^® ^(SPSS, Inc., Chicago, Illinois) was used for the data analysis.

## Results

A total of 145 patients were intravenously injected with ^18^F-FDG in anticipation for proceeding to the operating room on that same day for surgical exploration, biopsy, and possible resection of a known or suspected malignancy. This consisted of 98 females and 47 males. There were 134 Caucasian, eight African-American, and three Asian patients. Mean patient age was 57 (± 12, range 21-83) years. For all 145 participating patients, mean same-day ^18^F-FDG injection dose was 15.1 (± 3.5, range 4.6-26.1) mCi.

Of those 145 patients, a preoperative patient diagnostic PET/CT scan was done prior to the date of their anticipated ^18^F-FDG-directed surgery in 90 patients. In the remaining 55 patients, a preoperative patient diagnostic PET/CT scan was done on the same day as the anticipated ^18^F-FDG-directed surgery. For those 90 patients having their preoperative patient diagnostic PET/CT scan done prior to the day of their anticipated ^18^F-FDG-directed surgery, mean ^18^F-FDG injection dose for their prior preoperative patient diagnostic PET/CT scan was 14.3 (± 1.5, range 8.4-16.5) mCi, time from ^18^F-FDG injection to the preoperative patient diagnostic PET/CT scan was 80.6 (± 18, range 43-179) minutes, and mean duration of time from preoperative patient diagnostic PET/CT scan to the date of the anticipated ^18^F-FDG-directed surgery was 24 (± 18, range 1-92) days. For those 55 patients undergoing a same-day preoperative patient diagnostic PET/CT scan, mean same-day ^18^F-FDG injection dose was 16.4 (± 2.4, range 11.7-21.8) mCi and mean time from same-day ^18^F-FDG injection to their same-day preoperative patient diagnostic PET/CT scan was 73 (± 9, range 53-114) minutes.

Among the 145 patients intravenously injected with ^18^F-FDG in anticipation for proceeding to the operating room on that same day for surgical exploration, biopsy, and possible resection of a known or suspected malignancy, 142 were eventually taken to surgery on that same day. However, three of the same-day preoperative patient diagnostic PET/CT scans led to the cancellation of the anticipated surgical procedure. In two of these cases, same-day preoperative patient PET/CT scan demonstrated inoperable, widespread metastatic disease. In the other case, there was interval complete resolution of ^18^F-FDG avidity seen at the time of same-day preoperative patient diagnostic PET/CT scan as compared ^18^F-FDG avidity seen on a previously performed patient PET/CT scan.

Intraoperative gamma probe assessment was performed on 141 patients taken to the operating room for ^18^F-FDG-directed surgery. In the one case in which intraoperative gamma probe assessment was not performed, upon initial surgical exploration, it was noted that diffuse carcinomatosis was present, and resultantly, further surgical intervention was aborted. Mean time from same-day ^18^F-FDG injection to intraoperative gamma probe assessment was 286 (± 93, range 176-532) minutes.

WSRS were imaged by clinical PET/CT specimen scanning and micro PET/CT specimen scanning scan in 109 cases and 32 cases, respectively. Mean time from same-day ^18^F-FDG injection to scanning of WSRS by clinical PET/CT specimen scanning and micro PET/CT specimen scanning was 389 (± 148, range 86-741) minutes and 458 (± 97, range 272-656) minutes, respectively.

RDT were imaged by clinical PET/CT specimen scanning and micro PET/CT specimen scanning in 33 cases and 22 cases, respectively. Mean time from same-day ^18^F-FDG injection to scanning of RDT by clinical PET/CT specimen scanning and micro PET/CT specimen scanning was 619 (± 119, range 253-846) minutes and 661 (± 117, range 433-835) minutes, respectively.

SRDT pieces were imaged by clinical PET/CT specimen scanning and micro PET/CT specimen scanning in 49 cases and 26 cases, respectively. Mean time from same-day ^18^F-FDG injection to scanning of SRDT pieces by clinical PET/CT specimen scanning and micro PET/CT specimen scanning was 674 (± 186, range 299-1068) minutes and 752 (± 127, range 499-976) minutes, respectively. Tissue counting of individual SRDT pieces was performed on an automatic gamma well counter in 45 cases. Mean time from same-day ^18^F-FDG injection to performance of tissue counting of the SRDT pieces was 761 (± 187, range 324-1114) minutes.

A same-day postoperative patient diagnostic PET/CT scan was performed in 62 cases. Mean time from same-day ^18^F-FDG injection to the same-day postoperative patient diagnostic PET/CT scan was 516 (± 134, range 178-853) minutes.

Among the 142 patients who were taken to the operating room for ^18^F-FDG-directed surgery, 120 patients were found to have histologic confirmation of malignancy within their surgically resected specimens, 21 patients were found to have only benign pathology within their surgically resected specimens that were associated with their ^18^F-FDG-avid lesion(s) which were originally seen on their preoperative patient PET/CT scan, and one patient was intraoperatively found to have no detectable 18F-FDG-avid lesion (as well as no signs of disease on intraoperative ultrasound or on intraoperative visual inspection and palpation). Fourteen different histologic tumor types were represented within surgically resected specimens removed at the time of ^18^F-FDG-directed surgery from among the 120 patients with histologic confirmation of malignancy (Table 2). The three most common histologic tumor types found within surgically resected specimens were colorectal carcinoma (n = 66, 55.0%), breast carcinoma (n = 12, 10.0%), and lymphoma (n = 11, 9.2%). The region of localization of these 14 histologic tumor types among the 120 patients with histologic confirmation of malignancy was the abdomen and/or pelvis region in 87 cases (72.5%), the head and neck region in 12 cases (10.0%), the extremity, axillary, or inguinal regions in 12 cases (10.0%), and the chest region in 9 cases (7.5%).

**Table 2:** Fourteen histologic tumor types represented within the surgically resected specimens removed at the time of ^18^F-FDG-directed surgery from among the 120 patients with histologic confirmation of malignancy.

Histologic tumor type	Number (%) of patients
Colorectal carcinoma	66 (55.0%)

Breast carcinoma	12 (10.0%)

Lymphoma	11 (9.2%)

Ovarian carcinoma	7 (5.8%)

Head and neck squamous cell carcinoma	6 (5.0%)

Thyroid carcinoma	3 (2.5%)

Lung carcinoma	3 (2.5%)

Endometrial carcinoma	3 (2.5%)

Cervical carcinoma	2 (1.7%)

Melanoma	2 (1.7%)

Plasmacytoma	2 (1.7%)

Urothelial carcinoma	1 (0.8%)

Sarcoma	1 (0.8%)

Eccrine porocarcinoma	1 (0.8%)

## Discussion

The aim of this paper was to comprehensively describe the specific methodology utilized in our single-institution cumulative retrospective experience with a multimodal imaging and detection approach to ^18^F-FDG-directed surgery for patients with known or suspected malignancies. It is our belief that the information presented herein indicates that our multimodal imaging and detection approach to ^18^F-FDG-directed surgery for patients with known or suspected malignancies, utilizing a same-day ^18^F-FDG injection dose, is feasible from both a technical and a logistical perspective. This is evident from our ability to accomplish this coordination of services by the surgeon, radiologist, and pathologist in a same-day fashion. Such an integrated approach has the potential for allowing for: (1) real-time intraoperative staging of the extent of disease, (2) real-time intraoperative surgical planning and execution of the necessary and most appropriate operation, determination of the extent of surgical resection, and determination of the completeness of surgical resection, (3) real-time pathologic evaluation of intact surgical resected specimens for the confirmation of completeness of surgical resection and for surgical margin assessment, and (4) real-time pathologic evaluation of diagnostically biopsied tissues for confirmation of correctness of tissue diagnosis [32]. Since this paper purely represents a comprehensive description of the specific methodology utilized in our single-institution cumulative retrospective experience with a multimodal imaging and detection approach to ^18^F-FDG-directed surgery, and since this paper does not detail the specific cumulative results amassed from these 145 individual cases, it is the future plan of our current authorship to subsequently and systemically report upon many different aspects of our results, including analysis of image quality and sustainability of image quality seen on same-day preoperative patient diagnostic PET/CT imaging as it compares to same-day postoperative patient diagnostic PET/CT imaging, clinical PET/CT specimen imaging, micro PET/CT specimen imaging, automatic gamma well counter specimen activity, and intraoperative gamma probe assessment, as well as to report upon future clinical relevance, future clinical applications, and methodological limitations of this multimodal imaging and detection approach to ^18^F-FDG-directed surgery.

Whenever high-energy gamma photon emitting radiopharmaceuticals, such as ^18^F-FDG, are considered for routine use in the operating room environment, it is of importance to address the issue of radiation safety. In this specific regard, we have previously evaluated the occupational radiation exposure incurred by intraoperative and perioperative personnel involved during ^18^F-FDG-directed surgery cases [33]. In a comprehensive evaluation of 10 actual ^18^F-FDG-directed surgery cases, in which a mean dose of 18.9 mCi of ^18^F-FDG was intravenously injected at a mean time of 142 minutes prior to surgery, the resultant mean deep dose equivalent per case for the surgeon, anesthetist, scrub technologist, postoperative nurse, circulating nurse, and preoperative nurse was 164, 119, 92, 63, 54, and 48 μSv, respectively. Based upon the established annual occupational exposure limit for adults within the United States of a total effective dose equivalent of 50,000 μSv, as defined by the United States Nuclear Regulatory Commission [51], the estimated number of ^18^F-FDG-directed surgery cases per year and the estimated number of hours of exposure per year that could be theoretically incurred by the surgeon, anesthetist, scrub technologist, postoperative nurse, circulating nurse, and preoperative nurse were 305 cases and 820 hours, 420 cases and 1020 hours, 543 cases and 2083 hours, 794 cases and 1471 hours, 926 cases and 2941 hours, and 1042 cases and 602 hours, respectively. This data clearly illustrates that the absorbed radiation dose received by both intraoperative and perioperative personnel involved in ^18^F-FDG-directed surgery cases is relatively low per case and allows for all such personnel to participate in multiple cases and still remain well below regulatory standards set for occupational radiation exposure limits.

## Conclusions

In summary, our multimodal imaging and detection approach to ^18^F-FDG-directed surgery for patients with known or suspected solid malignancies is technically and logistically feasible. It can be accomplished with coordination of services provided by the surgeon, radiologist, and pathologist in a same-day fashion. This integrated approach has the potential for allowing for real-time intraoperative staging, surgical planning and execution, and determination of the completion of surgical resection.

## Competing interests

The authors declare that they have no competing interests.

## Authors' contributions

**SPP **was the principle investigator that supervised all aspects of this project. He was involved in study design, data collection, database construction, and data analyses. He organized, wrote, and edited all aspects of this manuscript. **NCH **supervised PET/CT imaging for this project, and was involved in study design and data collection. He was involved in writing and editing this manuscript. **DAM **supervised the day-to-day activities of this project and was involved in study design, data collection, database construction, and data analyses. He was involved in writing and editing this manuscript. **AZC**, **JRG**, **EEB**, **CMM**, and **MPK **were involved in data collection and database construction and edited this manuscript. **CLH **was the pathologists who were involved in reading and reviewing of the histopathology for many of the cases contained within this project, and edited this manuscript. **MVK **and **EWM **were the two most senior physicians/advisors involved in this project and were involved in study design and in editing this manuscript. All of the authors have read and approved the final version of this manuscript.
